# HOP'N after-school project: an obesity prevention randomized controlled trial

**DOI:** 10.1186/1479-5868-7-90

**Published:** 2010-12-13

**Authors:** David A Dzewaltowski, Richard R Rosenkranz, Karly S Geller, Karen J Coleman, Gregory J Welk, Tanis J Hastmann, George A Milliken

**Affiliations:** 1 Department of Kinesiology, Kansas State University, Manhattan, KS 66506, USA; 2 Department of Human Nutrition, Kansas State University, Manhattan, KS 66506, USA; 3 Research and Evaluation, Southern California Permanente Medical Group, Pasadena, CA 91101, USA; 4 Department of Kinesiology, Iowa State University, Ames, IA 50011, USA; 5 Department of Statistics, Kansas State University, Manhattan, KS 66506, USA

## Abstract

**Background:**

This paper reports the primary outcomes of the Healthy Opportunities for Physical Activity and Nutrition (HOP'N) after-school project, which was an effectiveness trial designed to evaluate the prevention of childhood obesity through building the capacity of after-school staff to increase physical activity (PA) and fruit and vegetable (FV) opportunities.

**Methods:**

We conducted a three-year, nested cross-sectional group randomized controlled effectiveness trial. After a baseline assessment year (2005-2006), schools and their after-school programs were randomized to the HOP'N after-school program (n = 4) or control (n = 3), and assessed for two subsequent years (intervention year 1, 2006-2007; intervention year 2, 2007-2008). Across the three years, 715 fourth grade students, and 246 third and fourth grade after-school program participants were included in the study. HOP'N included community government human service agency (Cooperative Extension) led community development efforts, a three-time yearly training of after-school staff, daily PA for 30 minutes following CATCH guidelines, a daily healthful snack, and a weekly nutrition and PA curriculum (HOP'N Club). Child outcomes included change in age- and gender-specific body mass index z-scores (BMIz) across the school year and PA during after-school time measured by accelerometers. The success of HOP'N in changing after-school program opportunities was evaluated by observations over the school year of after-school program physical activity sessions and snack FV offerings. Data were analyzed in 2009.

**Results:**

The intervention had no impact on changes in BMIz. Overweight/obese children attending HOP'N after-school programs performed 5.92 minutes more moderate-to-vigorous PA per day after intervention, which eliminated a baseline year deficit of 9.65 minutes per day (p < 0.05) compared to control site overweight/obese children. Active recreation program time at HOP'N sites was 23.40 minutes (intervention year 1, p = 0.01) and 14.20 minutes (intervention year 2, p = 0.10) greater than control sites. HOP'N sites and control sites did not differ in the number of FV offered as snacks.

**Conclusions:**

The HOP'N program had a positive impact on overweight/obese children's PA and after-school active recreation time.

**Trial registration:**

NCT01015599.

## Introduction

Over the last 10 years, school-based interventions aimed at preventing obesity have shown limited positive outcomes [1]. These disappointing findings may be due in part to the difficulties of implementing interventions in school settings, where competing demands for time have made it difficult to add anything other than academics to the school day [2]. After-school programs may offer promise for obesity prevention programming because there is more available time with fewer bureaucratic obstacles and curricular inflexibilities [3]. Enrollment in after-school programs has dramatically increased over the last twenty years [4]. The U.S. Department of Education estimated that 37.7% of all children in grades kindergarten (age 5-6) through 8 (age 13-14) participated in some form of organized after-school activity at least once per week [5].

Recently, several interventions have been developed to target after-school programs to promote physical activity (PA) and healthful eating [3,6,7]. Some studies have provided frequent PA [8], others have focused on providing children with theory-based PA and healthful eating skill-building experiences [9], and some have attempted to combine frequent, daily PA with skill-building sessions [10].

The Healthy Opportunities for Physical Activity and Nutrition (HOP'N) After-School Project takes a new approach to promoting PA and healthful eating by building the capacity of existing after-school programs to create healthy environments. This approach combines community level development, organizational level staff training, after-school program level environmental change, and skill building curriculum activities. Building effective multilevel obesity prevention practices into existing after-school programming may increase the likelihood that the program can be adopted and sustained with limited community resources [11].

This paper presents the main outcomes of a group randomized trial designed to evaluate the effectiveness of the HOP'N After-School Program for preventing obesity in children attending after-school programs. Relative to children at the control after-school sites, we hypothesized that children participating in the HOP'N intervention would have 1) less increases in age- and gender- specific body mass index z-scores (BMIz) across the school year and this difference would be greater in overweight and obese children, and 2) greater moderate-to-vigorous physical activity (MVPA). These differences in BMIz and MVPA would be due to more observed after-school PA sessions and fruit and vegetable (FV) snack offerings at the HOP'N after school sites compared to control sites.

## Methods

### Setting, Design, & Procedures

In the fall of 2005, all schools (n = 8) participating in an after-school program alliance of the Lawrence Public School District, Douglas County Cooperative Extension Service, Lawrence Boys and Girls Club, and community partners were considered for inclusion in the study. Of the eight sites, seven were invited and adopted the program. One site was not invited to participate because the after-school program was not on the elementary school grounds.

**Figure 1:**
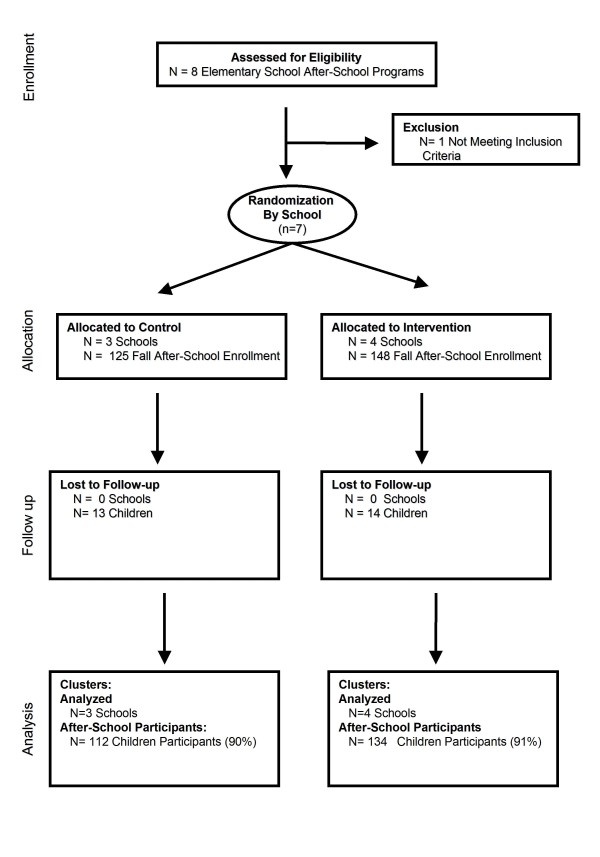
**CONSORT diagram of after-school participant flow through the trial**.

A three-year group-randomized controlled trial was conducted with random assignment at the school level after a baseline year of assessment (Figure 1). The study used a nested cross-sectional design with a baseline year (2005-2006), and two subsequent intervention years (2006-2007, 2007-2008). For each year of the study, new children in fourth grade and in after-school programs participated in the study. By using a "repeated cross-section" methodology the outcomes were tracked for the same places rather than for the same individuals [12]. If this study used a longitudinal design and attempted to follow students over three years, it is likely that participant dropout would have exceeded 30%. Although drop out would likely occur due to various reasons unrelated to the intervention, such as the family moving to another school district, change within an entire population can be best studied in a repeated cross section methodology when losses of participants due to movement or dropout are a concern [13].

After the baseline year, sites were stratified into two groups (High Socioeconomic Status (SES)/Low Diversity; Low SES/High Diversity) based on the percentage of students who qualified for free and reduced lunch, and the percentage of students who were nonHispanic white or of diverse race/ethnicity. Following stratification, within each matched group, the principle investigator used a random number generator to blindly randomize sites to the two-year HOP'N after-school intervention (n = 4) or to the control condition (n = 3). After baseline and the randomization procedure, the research team was not blind to the randomization.

In each study year, after-school participants in the fourth grade group underwent two data collection protocols. The first protocol was implemented in both the fall (Pretest) and the spring (Posttest) during after-school time. At least two research assistants traveled to the site and measured children on height and weight in a private setting. Height and weight were assessed while wearing light clothing and no shoes. Research assistants also administered a survey to children and sent a survey home to parents.

For the second protocol, after-school programs were observed by at least two research assistants on six days. Both control and intervention after-school sites were observed on a Monday, Tuesday, and Thursday (in random order) for no more than one day each month in the fall (three days) and in the spring (three days) semesters of each year. The second site visit protocol began with a research assistant attaching an ActiGraph accelerometer to each student's right hip via an adjustable elastic belt and recording the time of attachment and the identification number of the accelerometer and student. Then throughout the after-school day, one research assistant recorded the type of session offered to the children. The session activity was coded as academic, enrichment, recreational (active, nonactive), or snack. For sessions that offered PA, the other research assistant used the System for Observing Fitness Instruction Time (SOFIT) and categorized the active recreation sessions as either organized or free play. For sessions that offered a snack, all foods and beverages were recorded. At the end of the after-school program, or when a student left the early, the research assistant removed each accelerometer and noted time of removal.

Independent of these two data collection protocols, a third data collection effort involved all fourth graders (with informed parental consent) at each school regardless of their participation in the after-school program. Research assistants measured children's height and weight late in the spring semester of each year of the study during the school day. This fourth grade data set allowed us to examine the impact of HOP'N on the school as a whole. This school-wide sample also enabled us to determine whether the after-school program participants were representative of the overall school population [11]. The Institutional Review Board at Kansas State University approved all procedures in 2005.

### Participants

#### After-school participants

Children were included in the after-school sample if they were enrolled in the after-school fourth grade group, provided informed parental consent, and agreed to participate in the fall height and weight assessment. In the U.S., fourth graders are approximately nine years old. Because each after-school program, in addition to fourth graders, may have included third graders and some fifth and six graders to increase enrollment in the fourth grade group, we excluded children from the study if they were not in third or fourth grade and if they participated in the study in a previous year.

#### In-school participants

Similar to the after-school sample, each year a new group of children entered fourth grade and were eligible for participation. Children were included in the school sample (n = 716) if they were enrolled in a fourth grade school classroom in the fall of that year, had informed parental consent, and assented to participate in the height and weight assessment.

#### Intervention

Using Social Cognitive Theory [14] and an ecological developmental systems approach [7], the HOP'N intervention was designed to target the development of the skills and efficacy of adult leaders and children to build healthy after-school environments. The HOP'N intervention model included three levels: a community/government/human service agency (County Cooperative Extension), after-school staff training, and after-school program quality elements. The quality elements included an organized daily PA session for at least 30 minutes, a daily healthful snack that included a FV, and a weekly nutrition and PA education experience (HOP'N Club).

The first level of intervention targeted the development of the community/government/human service agency (County Cooperative Extension office) to coordinate improving after-school programs. The research team provided technical assistance to this agency, 5% salary for the Family and Consumer Science County Agent (who prior to the study coordinated the county nutrition education programs), and salary for a half-time Cooperative Extension assistant. The County Agent hired and supervised the Extension Assistant, conducted local community development work, sat on the school district's Wellness Council, and worked with school food service to improve the quality of the snack. The Extension Assistant coordinated the after-school staff training and delivered the HOP'N Club intervention at each site.

The Cooperative Extension office delivered the second level of intervention (after-school staff training) with the assistance of content expertise from the research team. This level of intervention included three staff training sessions per year (six sessions total), staff monthly meetings with the Extension Assistant, and continuous web support http://www.hopn.org. The training was modeled after the Healthy Places "performance community hub" where participants were encouraged to share and problem solve their implementation challenges [15,16]. Content for the first intervention year began with basic training on how to implement the HOP'N quality elements. Session content then progressed to goal setting, feedback, and problem solving strategies to achieve change in the after-school environment. Because there was high turnover in after-school staff, the sessions in the second intervention year paralleled year 1 in content.

For the third level of intervention, the after-school staff and the Extension Assistant implemented the HOP'N after school quality elements at each intervention site. The after-school program at each site was approximately 2.5 hours per day. Every day, staff had the goal to implement 30 minutes of organized PA following the CATCH Kids Club PA principles [2]. The project provided the CATCH Kids Club curriculum box [10] and PA equipment. Also, after-school program staff was directed to work with their school's food service to provide FV with every snack. In addition to this "*bottom-up approach*", the County Extension Office worked with the school district food service to achieve the same FV goal. To assist the program staff, the research team provided a list of healthy snack ideas and content expertise. Snacks were not purchased for the program.

Finally, also part of the third level of intervention, the HOP'N Club was a weekly social-cognitive-theory based curriculum delivered by the Cooperative Extension Assistant to each after-school intervention site for 60 minutes once a week. The curriculum was organized in a notebook form with weekly modules that included learning objectives, behavior change strategy goals, and implementation procedures and scripts. The HOP'N Club child behavioral goals were: Be physically active every day (30 minutes after-school, 60 minutes daily); eat FV at every meal or snack; drink less soda and juice drinks (drink water, no more than 1 can of soda or small cup daily); and cut back on TV and video games (no more than 2 hours a day; remove TV from bedroom). The behavioral goals were reviewed weekly and appeared on club cards, t-shirts, and other materials provided by the project. The first 15 sessions were delivered during the 2006 and 2007 fall semesters and targeted building children's competency to adopt and self-regulate behavior to meet the child behavioral goals. The next 14 sessions were delivered in the spring semesters of 2007 and 2008 and were designed to build children's skills and efficacy to influence their home and community environments.

An example of these skill-building activities was a "house hunt". Children took pictures of their home environment on a scavenger hunt, where they searched for physical environmental opportunities for healthful and unhealthful eating ("Go Foods" or "Whoa Foods") or opportunities for PA or sedentary behavior. Then, in "Cool Contracts" children selected a home environment change goal, role played how to ask their parents to participate in signing a contract to change the home environment, and completed a home environment change contract with their parents. Finally, after implementing environmental changes, the children again took pictures of their home and made a "HOP'N-at-Home" poster, which illustrated their home environmental changes. If parents did not want pictures taken in their home, the children completed the same poster experience by drawing pictures or cutting pictures out of magazines.

### Child Outcome Measures

#### Body Mass Index Z Scores (BMIz)

Height was measured using a portable stadiometer (Seca 214 Hamburg, Germany) and weight was measured using high precision digital scales (Seca Corp, Model 770, Hamburg, Germany) that were calibrated daily. Height and weight were measured twice and if the first two measurements differed by more than 1.0 centimeter or 0.1 kg, respectively, a third measure was taken, and the average of the two closest measures was used in the analyses. Unlike adults, BMI values in children do not provide an indication of overweight and obesity. Thus these values must be related to norm reference standards for growth by age and gender as recommended by the Centers for Disease Control and Prevention (CDC) [17]. This is done by using the CDC growth curves and obtaining a z-score for each BMI value. These z-scores are in turn related to the percentiles used to assess overweight and obesity. Participants were classified as "overweight" and "obese" using the age- and gender-specific 85^th ^and 95^th ^percentile BMI values for age and gender.

#### PA and Sedentary Behavior

Objectively monitored PA was assessed during after-school programs with ActiGraph GT1 M accelerometers (Shalimar, FL). The ActiGraph was programmed to record data every 30 seconds, and ''activity counts'' were processed using cut points developed by Freedson and colleagues [18] to estimate minutes of sedentary activity (< 50 counts per 30 seconds), light activity (≥ 50 counts to 3.9 METS), moderate activity (≥ 4 to 6.9 METS), and vigorous activity (≥ 7 METS) [19].

### After-School Environment and HE and PA Opportunities

Details about the observational system developed for HOP'N are available elsewhere [20]. Briefly, after-school programs were observed to determine if activities offered to children were academic, enrichment, recreational (active and non-active), or snack. A PA opportunity was defined as an active recreation session that involved any type of PA and was subcategorized as either organized or free play. Non-active recreation involved activities that were not designed to build skills and included activities such as board games, reading for fun, or computer use for fun. The active recreation sessions were objectively coded using the activity intensity categories of SOFIT to determine the time spent in MVPA [21]. All observers using SOFIT had agreements ≥ 80% for child PA intensity and ≥ 93% for lesson context.

A healthful snack opportunity was defined as offering a FV snack. The type of snack offered during after-school was observed and recorded for FV, total and fat calories, and carbohydrate grams. Nutritional content of the snack was evaluated using actual snack labels, company website nutrition information, and/or the USDA National Nutrient Database. Samples of snacks without labels were collected and weighed, and information was obtained from the USDA database by weight. Only FV offerings are reported here.

### Statistical Analyses

The impact of the intervention on the child outcome measures was analyzed using methods to adjust for the lack of independence of the data [13]. Student data were associated with other student data within school sites (i.e., intraclass correlation). To adjust for the clustered data structure, a mixed model three-level design structure (school, year, child) was used to analyze the after-school participant outcomes (fall to spring academic year change in BMIz, fall to spring academic year change in BMI, and accelerometer measured PA across the year) and fourth grade student outcomes (Spring BMIz, Spring BMI).

Separate analyses of covariance using PROC MIXED (SAS Version 9.1, SAS Institute Inc., Cary, NC) were conducted. Variables in each of these analyses were condition (HOP'N, Control), year (baseline, intervention yr 1, intervention yr 2), weight status (overweight/obese, normal), school stratification for randomization (High SES/Low Diversity, Low SES/High Diversity), grade, gender (boy, girl), race/ethnicity (white, nonwhite), and family SES (eligible for free and reduced lunch, not eligible). For the BMI and BMIz change score analyses, the fall (baseline) assessment was also a covariate. For PA, accelerometer monitoring time was additional covariate.

The study was originally powered to detect a .5 kg/m^2 ^difference in BMI between a sample size of 4 intervention and 4 control schools with a reduction in the detectable difference adjusting for age, ethnicity, and gender using 20 students per group. Assuming that the small after-school dropout from fall to spring was due to random factors, the after-school analyses examined the impact of HOP'N for those who initiated the program regardless of their level of attendance and dose of receiving the intervention. Comparisons of correlated response variables evaluating between condition and between year differences were evaluated at p < 0.05, two-tailed tests. Some alpha level adjustment for multiple tests should be made, but because of the complexity of the model there are not methods available to carry out such adjustments. A Bonferroni adjustment could be used but it would be too severe and would cover up possible meaningful comparisons where as an adjustment that would utilize the model and correlations would not [22].

The impact of the intervention on the after-school site PA session opportunities and FV snack offerings was analyzed using a non-parametric Mann-Whitney *U *test for each group comparison.

## Results

### Settings and Participant Reach and Representativeness

Figure 1 provides the schematic for setting and after-school participant enrollment. All seven sites that met inclusion criteria participated across the three-year study (100% adoption). A total of 531 children participated in the fourth grade after-school group across the three-year study. For these children, 411 (77%) had parental consent to participate and 371 (70%) participated in the Fall BMI assessment. At certain sites, the fourth grade group included third, fifth, and six graders. We excluded students if they had if they were not in third or fourth grade (N = 42) and if they were in fourth grade and had participated in the intervention in third grade (N = 56). Figure 1 illustrates 273 (51%) met our inclusion and exclusion criteria. For the after-school sample (n = 273), across all years of the study, 90% of control site children (n = 112) and 91% (n = 134) of intervention site students completed both the fall and spring assessments. A comparison between children participating in the fall with those present in the spring is depicted in Table 1.

**Table 1:** Pretest Characteristics of After-School Participants at Control or HOP'N Afterschool Programs

	Fall Pretest		Participants Completing Spring Posttest	
	Control	HOP'N	Control	HOP'N
Participants, n				
Baseline Yr	43	29	39	27
Intervention Yr 1	32	57	28	50
Intervention Yr 2	50	62	45	57
Total	125	148	112	134
Gender, % (n)				
Male	54 (68)	47 (70)	53 (59)	47 (63)
Female	46 (57)	53 (78)	47 (53)	53 (71)
S.E.S., % (n)				
Not Eligible	42 (51)	56 (80)	43 (48)	54 (70)
Free/Reduced	58 (70)	44 (63)	57 (63)	46 (59)
Ethnicity/Race, % (n)				
American Indian/Alaska Native	10 (13)	7 (11)	10 (11)	8 (10)
Asian	1 (1)	1 (1)	1 (1)	1 (1)
Black/African American	28 (35)	11 (16)	28 (31)	11 (14)
Hispanic/Latino	5 (6)	8 (12)	5 (6)	9 (12)
Native Hawaiian/Pacific Islander	1 (1)	1 (1)	1 (1)	1 (1)
White	53 (66)	71 (104)	54 (61)	71 (94)
Other	2 (2)	1 (2)	1 (1)	1 (1)
Age, Years (SD)	9.19 (0.66)	9.34 (0.65)	9.21 (0.66)	9.37 (0.63)
BMI Fall, kg/m^2 ^(SD)	18.85 (4.04)	18.87 (4.03)	19.00 (4.14)	18.98 (4.18)
BMI-Z Fall (SD)	0.62 (1.05)	0.65 (0.91)	0.64 (1.08)	0.66 (0.94)
Weight Status, % (n)				
Normal	65 (81)	66 (97)	63 (70)	64 (86)
Overweight	15 (19)	19 (28)	16 (18)	19 (25)
Obese	20 (25)	16 (23)	21 (24)	17 (23)
Overweight/Obese	35 (44)	34 (51)	38 (42)	33 (48)

Note. BMI = Body Mass Index in Kg/m^2^. BMIz is age and gender standardized.

The school fourth grade sample (n = 716) reached 86% of the total students enrolled each fall across the three years of the study. Fourth grade participants were 50% male, 71% nonhispanic white, 31% overweight or obese 15% obese, and 39% qualified for free and reduced lunch.

To examine representativeness of the data, we compared the fourth grade after-school students to demographic information reported by the schools in aggregate for all fourth graders attending during the fall semester of each year of measurement. After-school participants were similar to all fourth graders in terms of gender (50% boys and girls for both), but were more ethnically diverse with *lower *percentage of non-Hispanic white (after-school = 62%; School = 72%), and had a *higher *percentage of children with free/reduced lunch eligible status (after-school = 48%; school = 38%). Of all fourth grade students, after-school programs reached 32% across the study years.

### Child Body Mass Index

For after-school participants across the three years of study, Table 1 illustrates there were no differences in fall pretest BMI or BMIz scores between children in control after-school sites and children participating in HOP'N. Table 2 presents data for the change in BMI and BMIz scores across each school year. There were no differences between intervention and control sites. Although not significant, there was a trend for group differences in BMIz score changes (*p *= 0.11) in children who were initially overweight/obese. At control sites, BMIz scores increased by .4 units during the first intervention year, while overweight/obese children from the intervention sites did not change. This trend was not seen in the second year of the study.

**Table 2:** After-School Program Participants Adjusted Mean Differences (SE) by condition and pretest weight status.

Variable	Spring Posttest - Fall Pretest Adjusted Mean Change (SE)^2^						p^1^
	Control Sites			HOP'N Sites			
	Yr. B	Yr. 1	Yr. 2	Yr. B	Yr. 1	Yr. 2	
Body Mass Index (BMI)							.17
All Participants	0.3 (0.3)	0.9 (0.4)	0.2 (0.3)	0.7 (0.3)	0.1 (0.3)	0.1 (0.3)	
< 85 Percentile BMI	0.6 (0.3)	0.5 (0.4)	0.4 (0.3)	0.4 (0.4)	0.2 (0.3)	0.1 (0.3)	
≥ 85 Percentile BMI	0.0 (0.4)	1.3 (0.5)	0.1 (0.4)	1.0 (0.4)	0.1 (0.4)	-0.1 (0.3)	
BMI Z-Score (BMIz)							.11
All Participants	0.1 (0.1)	0.1 (0.1)	0.0 (0.1)	0.1 (0.1)	-0.1 (0.1)	-0.1 (0.1)	
< 85 Percentile BMIz	0.1 (0.1)	-0.1 (0.1)	0.1 (0.1)	0.0 (0.1)	-0.1 (0.1)	-0.2 (0.1)	
≥ 85 Percentile BMIz	0.0 (0.1)	0.4 (0.2)	-0.1 (0.1)	0.2 (0.1)	0.0 (0.1)	-0.1 (0.1)	

Note. BMI = Body Mass Index in Kg/m^2^. BMIz is age and gender standardized.

Yr. B = Baseline Year. Yr. 1 = Intervention year 1. Yr. 2 = Intervention year 2.

^1^Mixed model ANCOVA weight status by condition by year interaction test.

^2^Mixed model ANCOVA means adjusted for school random effect and condition, strata, year, demographic variables and demographic variable interactions with condition, strata, and year fixed effects. Demographic variables included S.E.S., race/ethnicity, and grade for BMI and BMIz. BMI means were also adjusted for age and gender.

For the school-wide fourth grade sample, there was also no difference in BMIz at the end of the baseline year and no changes over years between control schools (baseline mean = 0.82, SE = 0.17; yr 1 mean = 0.91, SE = 0.26; yr 2 mean = 1.09, SE = 0.19) and intervention schools (baseline mean = 1.05, SE = 0.19; yr 1 mean = 0.80, SE = 0.21; yr 2 mean = 1.05, SE = 0.14). Similarly, there were no differences at baseline and there were no differences in changes in BMI between control schools (baseline mean = 18.64, SE = 0.54, yr 1 mean = 19.96, SE = 1.11; yr 2 mean = 20.07, SE = 0.65) and intervention schools (baseline mean = 19.15, SE = 0.44, intervention yr 1 mean = 18.90, SE = 0.72, intervention yr 2 mean = 19.75, SE = 0.45).

### Child PA During After-School Time

**Figure 2:**
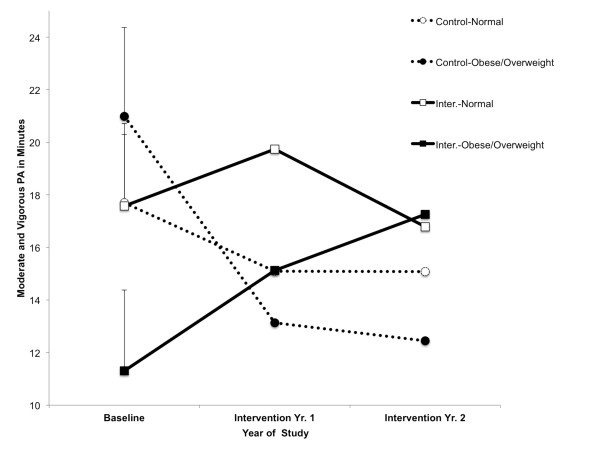
**Influence of HOP'N After School Program on moderate-to-vigorous physical activity by weight status**. Upper bars for the baseline year are standard errors.

Table 3 presents the findings for accelerometer PA for HOP'N and control after-school sites. HOP'N sites overweight/obese children accelerometer measured MVPA changed differently over years compared to control site overweight/obese children (*F *(4, 173) = 2.58, *p *=0.04) (see Figure 2). The MVPA for overweight/obese children at control sites *decreased *over years by 9.65 minutes per day (*t *= 2.10, *p *= 0.05, CI = 0.13 to 16.93), while MVPA for overweight/obese children at intervention sites approached a significant *increase *of 5.92 min per day (*t *= 1.65, *p *= 0.10, CI = -13.00 to 1.17). Rather than leading to a significant difference between conditions, the significant interaction was the result of a baseline significant difference in MVPA for overweight/obese children attending control sites (20.98 min) compared to overweight/obese children attending intervention sites (11.33 min, *t *= 2.12, *p *= 0.04) to be not significant during intervention (intervention year 1, control = 13.13 min, intervention = 15.13 min: intervention year 2, control = 12.44 min, intervention = 17.25 min).

**Table 3:** Adjusted Accelerometer Physical Activity (SE) by Condition and Weight Status for After-School Participants (N = 246).

After School Session	Adjusted Means (SE)^1^						Condition*Weight Status p^2^
Variable	Control Sites			HOP'N Sites			
	Yr. B	Yr. 1	Yr. 2	Yr. B	Yr. 1	Yr. 2	
All After School Time (Mean Minutes)							
Sedentary							0.24
Normal	39.93 (4.43)	33.10 (4.90)	32.38 (4.31)	39.21 (4.30)	28.02 (4.00)	32.86 (3.67)	
Overweight/Obese	32.32 (5.05)	36.18 (5.81)	32.30 (4.92)	46.92 (4.70)	31.35 (4.82)	32.65 (3.92)	
Vigorous							0.34
Normal	7.17 (1.77)	4.59 (1.93)	6.39 (1.73)	5.92 (1.68)	6.25 (1.56)	7.07 (1.46)	
Overweight/Obese	7.34 (1.98)	2.29 (2.24)	3.63 (1.94)	2.45 (1.81)	3.33 (1.86)	5.99 (1.55)	
MVPA							0.04*
Normal	17.70 (3.04)	15.09 (3.30)	15.80 (3.00)	17.57**^b ^** (2.86)	19.74 (2.67)	16.79 (2.51)	
Overweight/Obese	20.98**^a ^** (3.40)	13.13 (3.80)	12.44^**c**^ (3.31)	11.33**^ab ^** (3.09)	15.13 (3.15)	17.25 (2.65)	
All Active Recreation Time (Percent Time)							
Sedentary							0.01
Normal	19.94 (4.70)	12.39 (5.20)	15.65 (4.57)	7.72**^b ^** (4.70)	10.98 (4.18)	14.54 (3.92)	
Overweight/Obese	14.73 (5.33)	23.13 (6.09)	14.48 (5.18)	19.11**^b ^** (5.20)	14.69 (5.04)	10.62 (4.14)	
Vigorous							0.26
Normal	17.48 (6.20)	15.48 (6.64)	23.90 (6.09)	18.12 (5.93)	18.61 (5.39)	23.62 (5.14)	
Overweight/Obese	17.70 (6.75)	8.57 (7.47)	13.96 (6.64)	12.78 (6.41)	8.16 (6.19)	21.44 (5.34)	
MVPA							0.13
Normal	42.91 (8.07)	41.84 (8.67)	51.14 (7.93)	50.87 (7.72)	48.36 (7.02)	48.97 (6.69)	
Overweight/Obese	45.09 (8.82)	28.74 (9.79)	43.29 (8.66)	41.23 (8.36)	36.13 (8.11)	51.19 (6.97)	
Organized Active Recreation Time (Percent Time)							
Sedentary							0.04*
Normal	22.49**^b^** (4.51)	11. 04**^b ^** (5.54)	7.16 (4.41)	4.11**^b ^** (4.89)	15.23**^c ^** (4.29)	11.75 (3.72)	
Overweight/Obese	12.72^**ab **^ (5.93)	27.0**^c ^** (7.13)	8.05**^c ^** (5.46)	18.77^**ab **^ (5.75)	14.93 (5.85)	11.51 (4.16)	
Vigorous							0.23
Normal	11.92 (6.21)	14.83 (6.78)	22.78 (5.97)	22.56 (5.97)	20.04 (5.46)	25.45 (5.15)	
Overweight/Obese	15.24 (6.70)	1.73 (7.74)	12.65 (7.31)	17.42 (6.57)	12.55 (6.42)	22.65 (5.37)	
MVPA							0.38
Normal	33.66 (7.04)	35.35 (8.04)	49.28 (7.80)	46.17 (6.92)	36.49 (7.88)	35.37 (6.05)	
Overweight/Obese	36.93 (7.90)	20.03 (7.91)	44.15 (9.00)	29.50 (7.01)	47.89 (5.83)	44.33 (6.34)	

Note. Yr. B = Baseline Year. Yr. 1 = Intervention year 1. Yr. 2 = Intervention year 2.

^1^Mixed model ANCOVA Means adjusted for school random effect and condition, strata, year, monitor wearing time, demographics (gender, S.E.S., race/ethnicity, age, and grade) and demographic interactions with condition, strata, and year fixed effects.

^2^Mixed model ANCOVA weight status by condition by year interaction test. A significant difference reflects differences in the response variable due to baseline line weight status and the intervention.

* Significant at p < .05.

**^a^**Significant difference (p < .05) between conditions(intervention, control) at same time period within weight status groups (normal, overweight/obese).

**^b^**Significant difference (p < .05) between weight status groups (normal, overweight/obese) at same time period within conditions (intervention, control).

**^c^**Significant difference (p < .05) from prior time period within weight status groups(normal, overweight/obese)

At intervention sites, overweight/obese children performed less MVPA (11.33 min) than normal weight children (17.57 min) at baseline (*t *= 2.60, *p *= 0.01). This difference was smaller during intervention year 1 (*t *= 1.85, *p *= 0.06; overweight = 15.13 min; normal = 19.74 min) and was eliminated during intervention year 2. The increase in MVPA for overweight/obese children at invention sites (baseline, 11.33 min; intervention year 1 = 15.13 min; intervention year 2 = 17.25 min) approached significance (*t *= -1.65, *p *= 0.10).

There was a significant condition by years interaction in the proportion of sedentary activity during both active recreation time (*F*(4, 169) = 3.34, *p *= 0.01), and organized active recreation time (*F*(4, 150) = 2.56, *p *= 0.04). Overweight/obese children at intervention sites were significantly more sedentary during active recreation time than normal weight children during the baseline year (overweight/obese = 19%; normal = 8%), but this difference did not exist during the first intervention year (overweight/obese = 15%, normal = 11%) or the second intervention year (overweight/obese = 11%; normal = 15%). For normal weight children, during the baseline year there was a significant difference in the percentage of time spent in sedentary activity at control sites compared to intervention sites (23% vs. 4%; *t *= 2.76, *p *= .001). During the first and second intervention year no differences were observed.

There were no significant differences in the proportion of time spent in light, moderate, vigorous PA or MVPA time during active recreation time (e.g., organized games and free play) between HOP'N and control sites. There were also no differences in activity levels when only organized active recreation time was examined.

### After-School HE and PA Opportunities

#### Active Recreation Time

There were no differences in the time spent in active recreation at baseline between HOP'N (mean = 48.0 min, SD = 27.4) and control sites (mean = 44.6 min, SD = 27.8). During intervention year 1, HOP'N sites (Mean = 51.5 min, SD = 26.8) spent significantly more minutes in active recreation (+23.4 minutes; *z *= -2.747, *p*= 0.006) compared to control sites (Mean = 28.1 min, SD = 17.6). During intervention year 2, HOP'N sites spent 14.2 more minutes than control sites in active recreation sessions; however, this difference was not statistically significant.

Minutes in other session categories were similar at baseline for snack (HOP'N mean = 16.3 min, SD = 7.4; Control mean = 13.5 min, SD = 5.9), academics (HOP'N mean = 30.5 min, SD = 25.8; Control mean = 26.0 min, SD = 19.9), non-active recreation (HOP'N mean = 14.4 min, SD = 17.3; Control mean = 11.2 min, SD = 19.2) and enrichment (HOP'N mean = 10.9 min, SD = 21.2; Control mean = 8.0 min, SD = 17.5) and during both intervention years.

#### Active Recreation MVPA Time

At baseline, the mean minutes spent per day in observed MVPA during active recreation time for HOP'N sites (17.9 min, SD = 10.7) and control sites (16.0 min, SD = 11.3) was similar. HOP'N sites had (*p* = 0.001) significantly more MVPA time during active recreation (18.7 min, SD = 9.5) than control sites (12.2 min, SD = 12.7) in intervention year 1. This significant difference was not maintained in year 2 of intervention (HOP'N mean = 15.9 min, SD = 9.6; control mean = 15.1, SD = 6.3) ( *p*= 0.80). At baseline the percent of time in MVPA observed during active intervention time for intervention sites was 56% (SD = 12.6%) and control sites was 52% (SD = 17.0). There was no difference during intervention year 1 (HOP'N mean = 52%, SD = 17.5; control mean = 54%, SD = 16.8; *p* = 0.54) and intervention year 2 (HOP'N mean = 59%, SD = 13.0; control mean = 55%, SD = 13.1; *p* = 0.46).

#### Healthful Snacks

There were no significant differences between intervention and control sites for FV servings offered as part of after-school snacks at baseline, nor during either intervention year 1 or 2.

## Discussion

The primary aim of this study was to evaluate the effectiveness of the Healthy Opportunities for Physical Activity and Nutrition (HOP'N) after-school program in preventing an increase in age- and gender-specific body mass index z-score (BMIz) across the school year. There was no difference in change in BMIz across the school year for children attending HOP'N sites compared to children attending control sites and there was no difference when children were categorized as overweight/obese. It is possible that HOP'N did not impact BMIz because, contrary to our hypothesis, there was no increase in BMIz for control schools across the three years of the study and no consistent increase in BMIz across the academic year for after-school program participants attending control sites.

We also hypothesized that the quality of the after-school environment would be improved, such that there would be an increase in PA and healthful eating opportunities at HOP'N sites compared to control sites. Contrary to our hypothesis, HOP'N sites did not increase in snack FV servings compared to control sites. Consistent with our hypothesis, HOP'N was effective at increasing PA opportunities, as intervention sites spent more time in active recreation than control sites. HOP'N site overweight/obese children had an increase in after-school MVPA of almost 6 minutes per day, which eliminated a baseline year deficit in MVPA compared to control site overweight/obese children. The intervention, which focused on instituting games that did not eliminate poor performers, appears to have motivated overweight/obese children but had little effect on normal weight children. Unfortunately, prior to intervention, baseline levels of active recreation offerings and MVPA were not the same at intervention and control sites even though these sites were part of the same organization. If this study had been able to randomly select a large number of after-school sites with various levels of active recreation offerings, then the impact of the intervention on a normal distribution of baseline active recreation offerings could have been evaluated. None-the-less, the 6 minute improvement in MVPA is substantial as this is 10% of a child's contribution to meeting a 60 minute per day MVPA public health guideline. Across study years, overweight/obese children attending control sites declined in MVPA. The factors contributing to this decline are unknown. However, if there were community influences that contribute to the decline in MVPA of control site overweight/obese children, it may be that the intervention buffered against these forces and the 6 minute increase is an underestimation of the intervention's impact.

In year 2, the HOP'N intervention was also effective in increasing the MVPA of overweight/obese children attending intervention sites compared to control sites, but not for normal weight children. This is one of the first studies to demonstrate that weight status moderated the effectiveness of an intervention to increase PA. The documented increase in MVPA for overweight/obese children has important implications, since overweight/obese children may be the most in need for school-based obesity prevention programs.

Other after-school intervention studies have shown improvements in obesity-related measures. For example, Georgia FitKid, demonstrated improvements in fitness and percent body fat for children who attended at least 40% of after-school sessions that offered 40 min of academic enrichment, healthy snacks, and 80 min of MVPA [23-27]. It may be that more PA is necessary than the 30 min standard of HOP'N to contribute to preventing obesity. A second reason for the difference in findings could be that the improvements in body fat found in the Georgia FitKid study were detected due to use of dual-energy x-ray absorptiometry, rather than BMIz. In fact, the children who participated in the FitKid intervention group actually increased in BMI compared to control.

Finally, HOP'N was a public health effectiveness trial that had limited direct investigator team contact and economic input into the community. The Georgia FitKid intervention included aspects of an efficacy trial, such that the team was involved in program delivery on site. Children were also eliminated from the analysis due to poor attendance, the program was free of charge, and the healthful snack, transportation, and staff received partial or total support from project funds. The after-school setting can be enhanced to facilitate obesity prevention; however, the challenge is in reaching children and motivating them to attend after-school programs regularly. Unlike efficacy models, our training model provides a method for program improvement that can be easily disseminated to improve the quality of existing after-school programs in community settings without considerable onsite face-to-face involvement by experts and without an investment in additional after-school staff.

The HOP'N intervention included the goal of offering FV with every snack. Unfortunately, HOP'N sites did not increase in snack FV servings compared to control sites. After-school studies such as Georgia FitKid and CATCH Kids Club have minimally intervened in after-school snack periods. However, to our knowledge neither of these programs reported changes in snack quality. Although it is beyond the scope of this paper to examine the processes that impacted HOP'N implementation, there is a need to investigate whether a more intensive multilevel intervention with greater economic incentives is necessary to create FV availability after-school.

Use of accelerometers to measure PA allowed us to examine the impact of the intervention on children's PA and sedentary behavior during after-school time, and to determine whether weight status moderated the effect of the intervention. During the baseline year at intervention sites, overweight/obese children engaged in less MVPA during after-school and spent a greater percentage of their active recreation time engaging in sedentary behavior compared to normal weight children. The HOP'N intervention was effective in eliminating these differences. Therefore, the intervention may have created an after-school environment that promoted PA in those children who were most in need. After-school group leaders were trained to replace games that included elimination (such as dodge ball) with games that allowed all children to continue participating regardless of their success. Although it has been shown that non-elimination games promote more PA than elimination games [28], during adult led activities in HOP'N there was a trend toward normal weight children increasing in sedentary behavior due to the intervention. Future research should investigate whether some games that promote PA for overweight/obese children may not be the best choice for all children.

HOP'N was one of the first group-randomized public health effectiveness trial studies conducted on after-school sites to prevent obesity. The randomized design and objective outcome measures were a major strength of this study. Although HOP'N was not effective in influencing BMIz, there were positive PA outcomes that suggested that HOP'N may provide a framework that could improve the quality of after school programming. HOP'N targeted building the skills and efficacy of adults to improve the quality of after-school programs through a continuous training model. Implementation of the training model was successful in changing the practices of after-school staff in their provision of PA options. Some success in implementing a similar training model has been documented for environmental change in middle schools [15]. Therefore, future investigations need to examine types of community development and staff training models that are effective in implementing environmental change in studies with more settings in more diverse contexts. Future investigations also need to illuminate whether individual difference factors, such as weight status, moderate the effectiveness of obesity prevention interventions for children.

## Conclusion

The HOP'N after-school program is a promising framework to promote healthful behavior by building the capacity of existing after-school programs to create healthy environments. The HOP'N program had a positive impact on after-school active recreation time and overweight/obese children's after-school MVPA. The HOP'N intervention, which combines community development and organizational level staff training, may be a training model that can be adopted and sustained by organizations leading obesity prevention efforts in after-school programs. In addition to community development and organization staff training, this model may benefit from a targeted food service staff intervention to influence healthful snack options in after-school settings.

## Competing interests

The authors declare that they have no competing interests.

## Authors' contributions

DAD was Principal Investigator for this study and was the lead writer on the manuscript. RRR and KSG contributed to developing the intervention and assisted with the development of the data collection procedures. KJC contributed to the development of the observation system, training protocol for observation staff, and data collection procedures. GJW analyzed the accelerometer data. TJH assisted with data collection and interpretation of findings. GAM designed the data analysis procedures. All authors critiqued and edited drafts of the manuscript and approved the submitted version.
